# Spot Weld Analysis With 2D Ultrasonic Arrays

**DOI:** 10.6028/jres.109.015

**Published:** 2004-04-01

**Authors:** A. A. Denisov, C. M. Shakarji, B. B. Lawford, R. Gr. Maev, J. M. Paille

**Affiliations:** University of Windsor, Windsor, Ontario N9B 3P4; National Institute of Standards and Technology, Gaithersburg, MD 20899; National Institute of Standards and Technology, Gaithersburg, MD 20899; University of Maryland, College Park, MD 20450; University of Windsor, Windsor, Ontario N9B 3P4; DaimlerChrysler, Auburn Hills, MI 48326

**Keywords:** least-squares, nondestructive analysis, spot weld, transducer, two-dimensional, ultrasonic

## Abstract

This paper describes a threefold method of testing the performance of an array-based ultrasonic tool for nondestructive testing of spot welds. The tool is described in its capabilities, use, and advantages over existing counterparts. Performance testing for and the results from carrying out the testing are described. The three performance testing methods include 1) the use of calibrated samples, 2) comparisons with actual spot-welds, and 3) a performance evaluation of the embedded fitting software. The test of the fitting software was carried out by a comparison of results with reference fits supplied by the National Institute of Standards and Technology.

## 1. Introduction

The problem of estimating the spot weld quality is an important component in quality control. The modern automobile, for example, includes several thousand spot welds that have to be reliable.

Current methods of spot-weld inspection include destructive peel testing, conventional ultrasonic testing, and testing with desktop acoustic microscopes. During the procedure of peel testing the weld is torn apart, and the size of the remaining pin-shaped weld is measured with a caliper. This is a time and labor consuming and labor intensive procedure, and the tested part is destroyed, which is not acceptable for inline testing. Standard ultrasonic testing, which nowadays is used in many plants, is performed with a single probe ultrasonic tester. The determination of the spot weld quality is made by the operator by analyzing an oscilloscope-type screen. This determination can be very subjective. Even attempting to estimate nugget size based on the amplitude of the received signal, the detailed information about the nugget is limited. In contrast, the desktop acoustic microscope (AM) generates a very detailed image of the nugget area by scanning with the high frequency ultrasonic lens over the surface of the welded area. It is currently the most accurate tool for nondestructive spot weld quality control. However, this desktop microscope solution is expensive, it is not portable, it cannot be used in the industrial environment, and it takes a long time to generate an image.

**Fig. 1:**
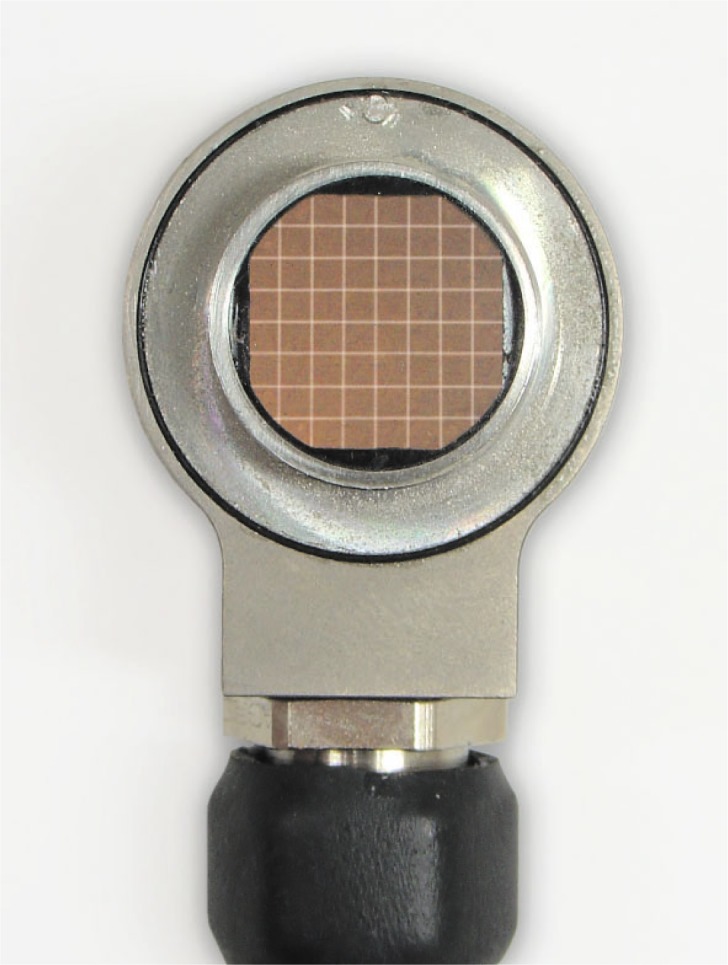
The matric transducer.

The 2D ultrasonic array as applied to spot weld testing is a new conception in ultrasonic testing [1–6]. Instead of mechanical scanning, it runs the signal across a matrix of small ultrasonic transducers. Each element in the matrix (Fig. 1) sends and receives signals independently, thus collecting the structural information on the part of the sample detected by the element. By combining the response from all the elements, one can build up an ultrasonic image of the internal structure at a specific depth. With this approach, the image acquisition time is much faster than when using a desktop microscope. (A matrix with 52 elements takes about 0.3 s to 0.7 s to visualize a 10 mm × 10 mm area).

The spatial resolution of 2D ultrasonic arrays is generally limited by the size of its elements. The process of the nugget diameter estimation, however, uses the whole ensemble of the elements; therefore, the effective precision in the diameter is higher than the spatial resolution [1]. But can the performance of this new method be quantified? How will one be able to evaluate the uncertainty of measurement using such a tool? The purpose of this paper is to study theoretical and practical aspects and limitations of nugget diameter estimation using a 2D ultrasonic array. Answering these questions is not straightforward. One key issue is the difficulty in obtaining known answers with which to compare detected results. One might consider measuring a weld and then breaking open the test part to measure the true weld size for comparison. But the destructive step itself might cause errors substantial enough to degrade the test significantly. Testing against results from a desktop microscope could be of value, but the question is then merely transferred to questions of the reliability of the desktop microscope measurements.

The purpose of this paper is to document means to answer these questions of performance when using 2D ultrasonic arrays. We study theoretical and practical aspects and limitations of nugget diameter estimation using this array-based method. Testing involves a combination of three methods: 1) The use of calibrated samples, 2) the use of a repeatability analysis on actual spot-welds, and 3) performance evaluation of the fitting algorithms used in the device.

The paper is organized as follows. We begin describing the instrument, its use and setup—particularly as used in the cases of our testing. The next section describes the means by which the instrument processes sampled information to report a location and diameter of the detected weld. Sections 4–6 detail the three-pronged testing method outlined above. We finally summarize our conclusions from the testing in Sec. 7.

## 2. Experimental Setup

The matrix transducer is a 2D array of ultrasonic transducers. The probe (Fig. 1) in our experiments consisted of 52 elements. The pitch of the matrix is (1.25 ± 0.05) mm with a small gap between elements that is (0.1 ± 0.05) mm thick.

**Fig. 2:**
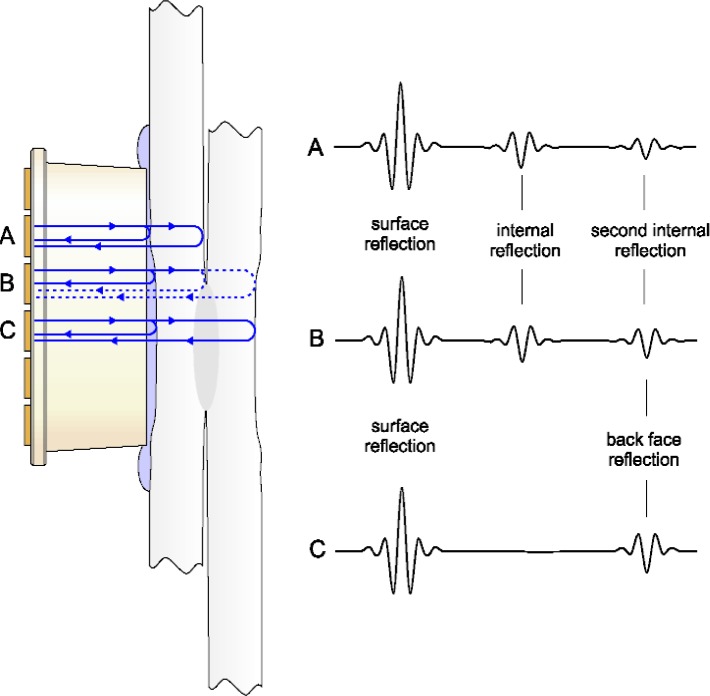
Wave paths (idealized) inside the sample (left) and the corresponding waveforms (right).

Each element of the matrix operates in the reflective mode, which means that the acoustic wave generated by the element is reflected back from the sample and detected by the same element. The wave is reflected from the surface of the sample and from the internal interfaces, thus providing the structural information on the part of the sample covered by the corresponding element. The transducer operates such that a sound wave reflected from a deeper defect arrives later in time than one reflected from a surface or subsurface defect (Fig. 2).

When inspecting spot welds, the presence of reflection from the area between the metal sheets indicates that the sheets are separated from each other; this corresponds to the non-welded area (Fig. 2A). If the metal is welded, there is no internal reflection, and only the reflection from the bottom of the second sheet can be detected (Fig. 2C). If the element covers an area that is partially welded, the resulting signal is a combination of the waveforms A and C.

**Fig. 3:**
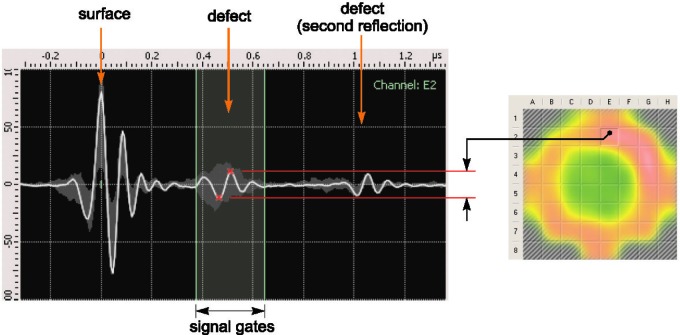
Building the image of the welded area.

**Fig. 4:**
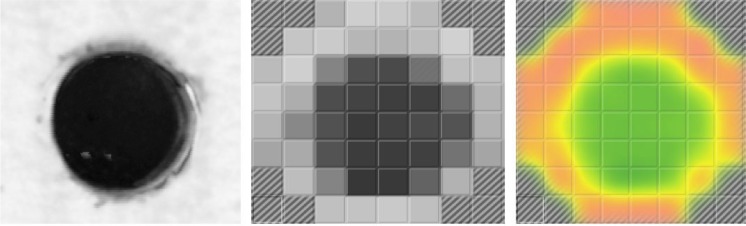
Image of the welded area obtained with the desktop acoustic microscope (left), matrix transducer without interpolation (center), and matrix transducer with interpolation (right). Each cell covers a 1 mm^2^ area.

To avoid the influence of neighbors, only one element of the transducer is active at the any instant of time. After the waveform is acquired, the controller activates another element, proceeding with the data acquisition until all the elements are processed [3]. The amplitudes of the waveforms from the acquired 52 scans are used to build an ultrasonic image of the welded area. The image can be displayed in grayscale or with color-coding by mapping the maximum amplitude from the predefined range (signal gates) to the brightness or color of the pixel. In Fig. 3, the green area in the center corresponds to the welded region, and the red area around it corresponds to the non-welded region. The visual appearance of the data can be improved using interpolation (Fig. 4). Several methods to increase the quality and stability of the image have been introduced in [1].

In this paper, we describe the algorithms for automatic estimation of the size of the welded area. This is the most important parameter in the spot weld quality assessment. For example, in the automotive industry a weld is considered good if the average diameter of the pin remaining after the destructive peel test exceeds a predefined minimum value.

## 3. Estimating the Spot Weld Size

The main goal of the algorithm is estimating the diameter of the welded area by analyzing the 8 × 8 matrix of amplitudes (though, as in Fig. 1, the circular transducer samples only a large subset of this square grid, without the corners). In addition to the size, the algorithm also reports the location of the center of the circle, which allows for visual feedback. The operator can instantly see if the algorithm seems to be performing as expected and if the estimation is reasonable.

A few comments should be made on the magnitudes of the amplitudes. Due to the variations in samples and measurement conditions, the range of values for these amplitudes cannot be fixed. We can assume only that the amplitudes of the elements that receive reflection from the non-welded area are higher than those for the other elements. Furthermore, due to the differences in the sensitivity of the elements and other factors, such as surface curvature, the relative sensitivity of each element can vary up to 20 %. On the other hand, there is a finite minimum in the measurable amplitude caused by the presence of noise in the system.

There is also the possibility of the disappearance of the signal for one or more elements due to bad measurement conditions, improper positioning of the transducer, or bad quality of the surface of the sample. Most of these issues are addressed by the algorithms that build the matrix of the amplitudes from the initial waveforms; hence, the signals from certain cells have weighting factors less than 1.0 in the analysis.

The scope of this paper deals with welds that are approximately round in shape. When the weld is not circular, say slightly elongated, the reported diameter should ideally be the diameter of a circle that has the same area of the weld shape. Welds that significantly differ from a round shape are considered outside the scope of this paper.

The algorithm for automatic diameter estimation takes a two-stage approach:

1. Estimating an approximate position of the center and diameter of the circle using the Hough transform [7].
2. Refining Measuring the diameter of the circle in polar coordinates.

The matrix of input amplitudes is first analyzed with a special version of a Hough transform algorithm that estimates the position and the diameter of the circle (*x_c_, y_c_, d*). The Hough transform algorithm returns the position of the center with a statistical standard uncertainty of 0.3 mm. Without refining the diameter, the error in the estimated size can be large—often exceeding 1.0 mm, which is not a sufficiently reliable classification of spot welds.

**Fig. 5:**
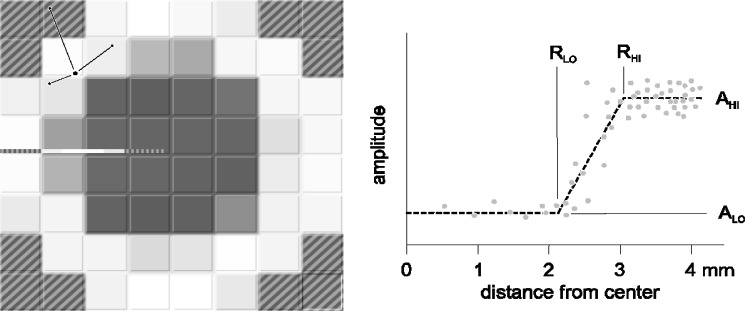
Building the image of the welded area. The unit of amplitude is arbitrary.

The diameter estimation is improved in the second stage. The location of the center is held fixed, and the size is refined based on the measured data. This is achieved by plotting the dependence of the amplitude vs. the distance from the center of the circle (Fig. 5).

The elements inside the welded area, in this case less than 2 mm from the center, have low amplitude corresponding to the noise level. Those elements more than 3 mm away from the weld correspond to the full reflection from the non-welded area. The rest of the amplitudes correspond to the partial coverage and have intermediate values. The size of the welded area can be determined from this data by fitting an S-shaped curve to these points. In practice, a good approximation can be obtained by fitting a curve composed of three straight lines (Fig. 5): two horizontal sections with amplitudes *A*_LO_ and *A*_HI_, corresponding to welded and non-welded areas, and a line which connects points (*R*_LO_, *A*_LO_)−(*R*_HI_, *A*_HI_). Therefore, this curve is specified by four variables that can be obtained using least squares fitting. The radius of the welded area can then be estimated as *R*_LO_ + (*R*_HI_− *R*_LO_) *f*, where *f* is the adjustment factor, which can be chosen experimentally.

With this approach, the result is automatically averaged. Note that if the shape of the welded area is not perfectly round or if the position of the center is not precise, in the first approximation, *R*_LO_ will decrease and *R*_HI_ will increase equally so that their average remains the same. This is why the precision of the coordinates of the center obtained in the previous stage is not very critical.

## 4. Performance Evaluation Using Calibrated Samples

**Fig. 6:**
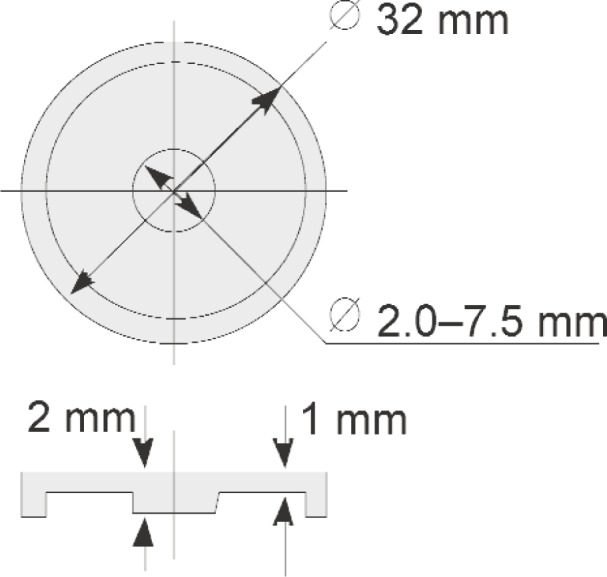
Calibration samples.

The first of three methods used to evaluate the performance of the matrix transducer tool, the resistance spot weld analyzer (RSWA), makes use of a set of manufactured calibration samples called “coupons.” These coupons simulate spot welds with nugget sizes ranging from 2.0 mm to 7.0 mm in 0.5 mm steps (Fig. 6).

Usage of such calibration samples allows testing the algorithm in near-ideal conditions. The maximum experimental repeatability and reproducibility of the algorithm can be tested without influence of other factors such as an error introduced by the peel test procedure (described later).

**Fig. 7:**
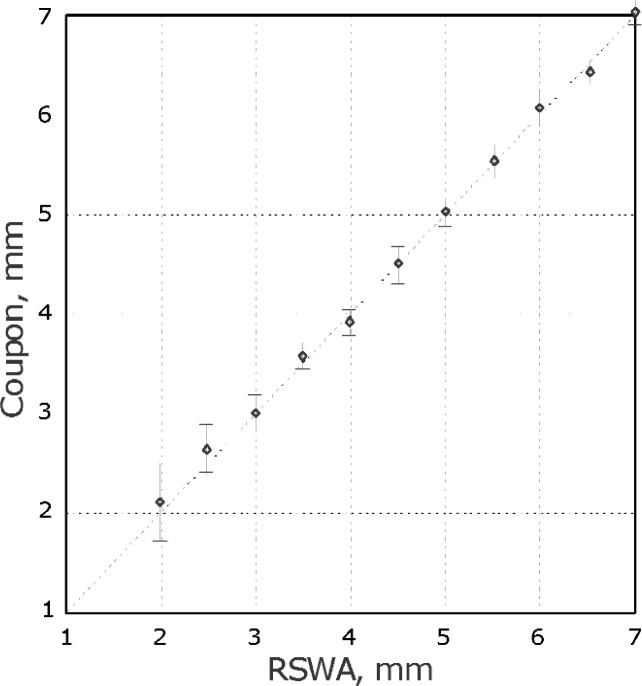
Experimental results.

The standard deviation in these tests ranges from 0.1 mm to 0.2 mm for small diameters and up to 0.5 mm for large diameters (see Fig. 7). Such degradation in precision can be attributed to the fact that for smaller diameters there are fewer elements in the neighborhood of the circle boundary. Since the amplitude of these elements is critical for size estimation, this measurement is more subjected to noise factors and the statistical uncertainty of the resulting diameter decreases.

## 5. Performance Evaluation by Studying Actual Spot Welds

A second means of assessing the performance of the matrix transducer involves comparisons with actual spot welds whose sizes are determined based on existing, reliable means. Correlation of measurements with peel tests is very important, since most industrial standards are based on the nugget size measured after the peel test. The major obstacle here is that the peel test itself is a subjective procedure. Depending on the materials used and the way the testing procedure is conducted, one can expect errors of up to 10 % to 20 %.

The uncertainty of the results of the desktop microscope, however, is much lower. When using calibration coupons, the desktop acoustic microscope demonstrates standard deviations of 0.05 mm or less. This is the precision of the coupons themselves. While comparisons were made against both the desktop microscope and values obtained through destructive analysis, we quantified the correlation of the RSWA measurements to the desktop microscope results, as they are deemed the more reliable reference values.

For the correlation test, a set of 30 welds with various nugget sizes was manufactured. A set of MS 6044A Galvaneal steel sheets with thicknesses of (0.75, 1.0, and 1.5) mm were welded with the Centerline VOLTZA^1^ weld gun producing nugget sizes from 1 mm to 6 mm.

**Fig. 8:**
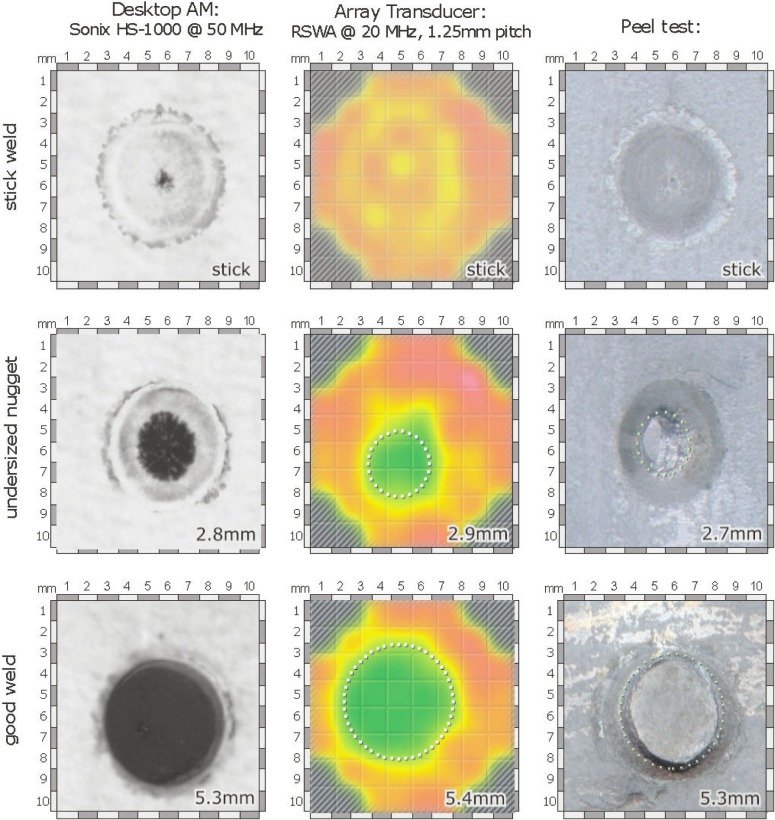
Comparing different methods for nugget size estimation.

Those samples were measured with the scanning acoustic microscope Sonix HS-1000 using a 50 MHz focused lens with an aperture of 12° (Fig. 8, left column). Then the same samples were measured with RSWA (Fig. 8, central column). Finally, optical images of peeled welds were obtained (Fig. 8, right column).

**Fig. 9:**
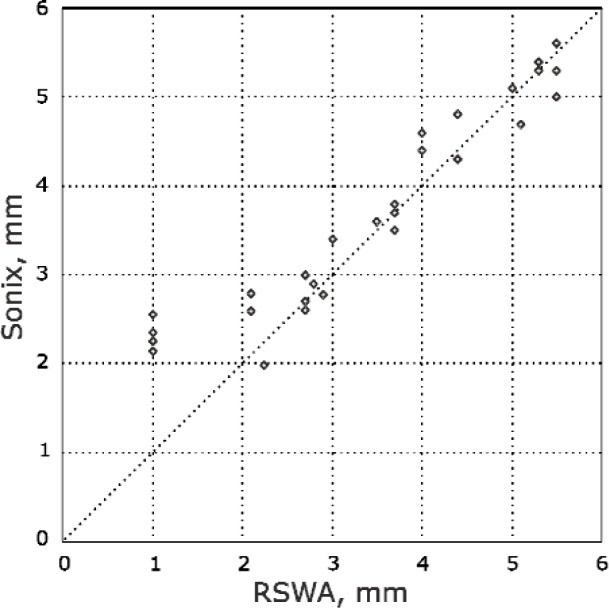
Experimentl results of the Sonix AM versus the RSWA.

Figure 9 demonstrates correlation between the desktop acoustic microscope and RSWA. From Fig. 9 the standard deviation for sizes larger than 3 mm is 0.3 mm. There is a discrepancy for smaller nuggets, but this does not create a problem in common contexts in which all weld sizes below 3 mm are uniformly classified as bad.

## 6. Performance Evaluation of the Fitting Software Using NIST Testing

A third means of evaluating the performance of the RSWA involves comparing its fitting algorithms using reference results. This testing isolates the software, and the results should be understood in that context. That is, the overall performance of the RSWA depends on the software as well as the data acquisition, so the overall tool performance is seen only when the software test results are combined with results from the previously described physical testing.

An inter-comparison was performed with algorithms generated at the National Institute of Standards and Technology (NIST). The NIST reference algorithms were created to provide an extremely reliable solution to the least squares fitting problem. The reference algorithms were not developed with speed or computational efficiency in mind; rather they were developed to establish a set of reference results that could be confidently relied upon for correctness.

The test plan is a simple, three-step process: 1) Generate data sets representative of real measuring situations, 2) submit the same data sets to both the software under test and the NIST reference algorithms for fitting, and 3) compare the fit results from the software under test with the reference fits and report on the deviations between the two.

### 6.1 Data Generation

**Fig. 10:**
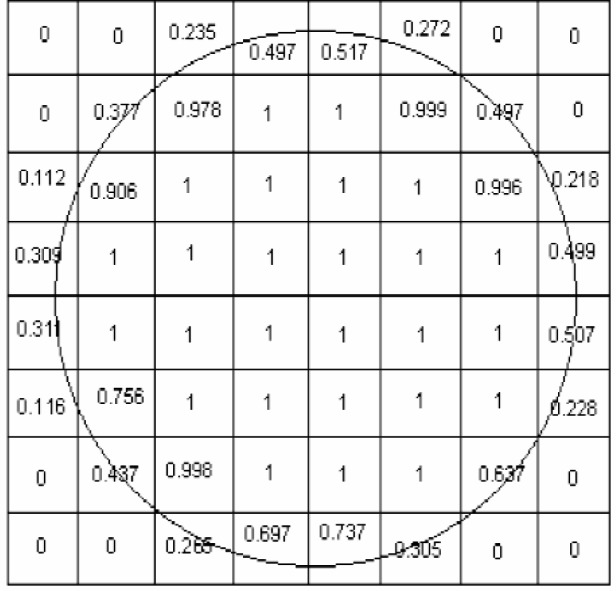
An example of a nominal weld (circle) and the proportion of area the circle covers for each cell.

A data set consists of 64 numbers, one corresponding to each cell in the 8-by-8 array. (The transducer does not gather data at three cells on each corner, but it is sufficient for testing the fitting algorithms to simply include them all.) The generation of these numbers involves a several-step process:

1. Determine a nominal circle position and size.
2. For each cell determine the proportion of the area of that cell that is covered by the circle (Fig. 10).
3. Add random noise to the value computed in the previous step (up to ±0.2 units).
4. Shift the data (so all entries are positive) by adding 0.2 to every element.
5. Scale data by multiplying by a factor randomly chosen between 0.5 and 0.8, in order to simulate the variation of signal strength from run to run.

Step 2 is nontrivial. We now present it in some detail, since the method to find the area of coverage is also used in the reference fitting algorithm. The mathematical problem is, given a circle and a cell, what proportion of the area of the cell is covered by the circle (Fig. 10).

**Fig. 11:**
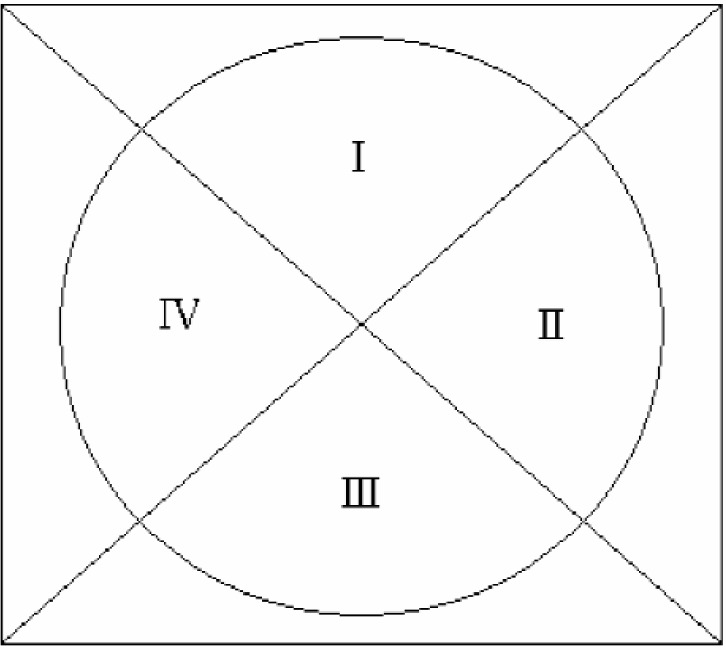
Division of the problem space into 4 regions.

We can assume without the loss of generality that the circle is centered at (0, 0), that the cell is at least partially in section I as depicted in Fig. 11, and that the cells in sections II, III, and IV are equivalent by symmetry. We also assume square cells having side length 1.0.

**Fig. 12:**
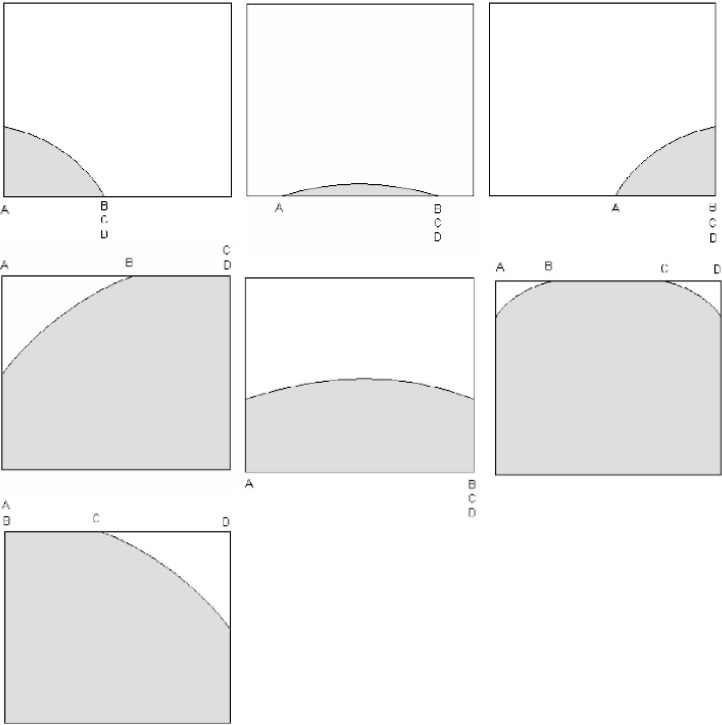
Cases of Partial Coverage of a Cell in Region I. This region is shown in Fig. 11.

If the cell is entirely inside or entirely outside the circle, the value is automatic (1 or 0, respectively). The clever use of rotations and translations reduces the remaining cases (of partial coverage) to those shown in Fig. 12.

In every one of the cases identified in Fig. 12, the formula for the area of the covered part of the 1 × 1 cell is given by

$$\int_{A}^{B}[g(x)-y_{\min}]dx+(C-B)+\int_{C}^{D}[g(x)-y_{\min}]dx,$$ (6.1)

where *g*(*x*) is the function of the circle of radius *R* centered at the origin, and *y*_min_ is the *y*-coordinate of the base of the cell. Note that when *A* = *B*, *B* = *C*, or *C* = *D* (as shown in Fig. 12), the corresponding component of Eq. (6.1) is zero. The integrals in Eq. (6.1) can be solved in closed form, making the calculation not computationally burdensome.

### 6.2 Reference Algorithm

The fitting problem at hand is as follows: Given an 8 × 8 matrix of values (generated as described in Sec. 6.1), find the circle (defined by its center and diameter) such that the areas of the cells covered by the circle most closely matches (in a least squares sense) the given values.

The goal of the reference algorithm is simply to provide reliable solutions to the problem. Matters of speed and generality are secondary. With that in mind, we developed our solution method as follows. We start by making the initial guess to be the original circle used in data generation (Sec. 6.1). To then search from there to the optimal circle, we chose two methods of search and then compared the results to ensure they were identical every time. The first minimization method was based on simulated annealing. This algorithm is known to find the minimum (in our case the least-squares solution) even when the absolute minimum is hidden among several nearby local minima. This is indeed the case in the problem at hand. The other method involved a multi-stage, adaptive brute force search. Neither method is efficient, computationally, but that is not a primary requirement for a reference algorithm. Both methods proved reliable, as they both returned the same results for all the data sets in this study.

### 6.3 Testing Procedure

In total, 50 data sets were generated and submitted to the software under test. We also submitted the reference results for the first five data sets. This was done to mimic actual practice. In actual practice, the matrix transducer is operated on about five physical reference samples in order to determine a scalar factor used by the system for an internal calibration. So likewise five random data sets were used in conjunction with their reference fits for this purpose. The remaining 45 data sets were used for the performance testing. The software under test imported the data, calculated the fits, and exported the fit results for comparison with the NIST reference results.

The data sets were generated with the diameter of the nominal generating circle (Sec. 6.1, step 1) chosen randomly in a range from 2.5 mm to 6.0 mm. The location of the center was also chosen randomly. We note that in some cases the center and radius were such that some (up to about 30 % of the area) of the circle lay outside the 64 cells. This corresponds to some real-world measurement situations.

### 6.4 Performance Evaluation Results

**Fig. 13:**
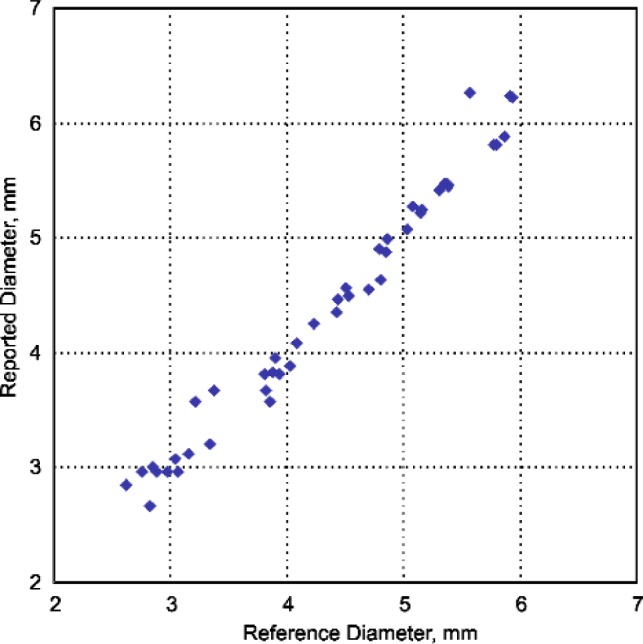
Correlation of diameters between reported and reference values.

The results of comparison between the fits reported by the software under test and the reference fits are summarized graphically in Fig. 13.

To quantify the deviations, we computed the mean and standard deviation in the fit diameter. The mean of the differences was 0.05 mm and the standard deviation was 0.17 mm. If the deviations were normally distributed about the mean, one would expect 68 % of the differences to fall within one standard deviation from the mean. In other words, we would expect a 68 % probability that for a given data set the reported diameter minus the reference diameter would be between −0.12 mm and 0.22 mm. Considering a zero mean, the standard deviation was 0.18 mm.

## 7. Conclusions

The performance test results from the three testing methods are consistent with one another, adding confidence to our overall performance evaluation. The first type of testing (using calibrated samples) showed a standard deviation of errors to be between 0.1 mm and 0.2 mm (though larger for the smallest diameters). The second method, using actual spot welds, yielded a standard deviation in the errors of 0.3 mm for all but the smallest diameters. The third test, performance evaluation of the embedded fitting algorithms, showed a standard deviation in the errors of 0.18 mm. The second method of testing accounts for more error sources than the other two, so it was expected that errors would be larger for that evaluation, which, in fact, was observed. As the evaluation method using actual spot welds most closely reflects real usage of the tool, the 0.3 mm standard deviation should be representative of actual usage, with the other two evaluation results adding confidence to its validity.

## Footnotes

1 Commercial equipment, instrumnets, or materials are identified in this paper to foster understanding. Such identification does not imply recommendation or endorsement by the National Institute of Standards and Technology, nor does it imply that the materials or equipment identified are necessarily the best available for the purpose.
